# The burden of bacterial antimicrobial resistance in Croatia in 2019: a country-level systematic analysis

**DOI:** 10.3325/cmj.2023.64.272

**Published:** 2023-08

**Authors:** Tomislav Meštrović, Kevin Shunji Ikuta, Lucien Swetschinski, Authia Gray, Gisela Robles Aguilar, Chieh Han, Eve Wool, Anna Gershberg Hayoon, Christopher J. L. Murray, Mohsen Naghavi

**Affiliations:** 1 Institute for Health Metrics and Evaluation, University of Washington, Seattle, WA, USA; 2 University North, University Centre Varaždin, Varaždin, Croatia

## Abstract

**Aim:**

To deliver the most wide-ranging set of antimicrobial resistance (AMR) burden estimates for Croatia to date.

**Methods:**

A complex modeling approach with five broad modeling components was used to estimate the disease burden for 12 main infectious syndromes and one residual group, 23 pathogenic bacteria, and 88 bug–drug combinations. This was represented by two relevant counterfactual scenarios: deaths/disability-adjusted life years (DALYs) that are attributable to AMR considering a situation where drug-resistant infections are substituted with sensitive ones, and deaths/DALYs associated with AMR considering a scenario where people with drug-resistant infections would instead present without any infection. The 95% uncertainty intervals (UI) were based on 1000 posterior draws in each modeling step, reported at the 2.5% and 97.5% of the draws’ distribution, while out-of-sample predictive validation was pursued for all the models.

**Results:**

The total burden associated with AMR in Croatia was 2546 (95% UI 1558–3803) deaths and 46 958 (28,033–71,628) DALYs, while the attributable burden was 614 (365–943) deaths and 11 321 (6,544–17,809) DALYs. The highest number of deaths was established for bloodstream infections, followed by peritoneal and intra-abdominal infections and infections of the urinary tract. Five leading pathogenic bacterial agents were responsible for 1808 deaths associated with resistance: *Escherichia coli*, *Staphylococcus aureus*, *Acinetobacter baumannii, Klebsiella pneumoniae*, and *Pseudomonas aeruginosa* (ordered by the number of deaths). Trimethoprim/sulfamethoxazole-resistant *E coli* and methicillin-resistant *S. aureus* were dominant pathogen-drug combinations in regard to mortality associated with and attributable to AMR, respectively.

**Conclusion:**

We showed that AMR represented a substantial public health concern in Croatia, which reflects global trends; hence, our detailed country-level findings may fast-track the implementation of multipronged strategies tailored in accordance with leading pathogens and pathogen-drug combinations.

Antimicrobial resistance (AMR) belongs to the most pressing public health hazards worldwide and demands the commitment of all countries in determining priorities and framing adequate policies (1). The most wide-ranging global assessment of AMR burden thus far (2) showed 4.95 million deaths associated with resistance of bacteria to antibiotics, and 1.27 million deaths attributable to resistance in 2019 - and revealed an unanticipated magnitude of the threat in Europe as well. Moreover, the Organization for Economic Co-operation and Development forecasts that the expenses linked to AMR complications may surge to 3.5 billion US dollars per year among 2.4 million people in Europe, Australia, and North America during the next thirty years if not addressed properly (3).

Croatia has been aware of the importance of this hazard for several decades. AMR surveillance in this country was inaugurated in 1996 with the establishment of the Croatian Committee for Antibiotic Resistance Surveillance at the Croatian Academy of Medical Sciences (4). This step facilitated the country’s efforts to join the European Antimicrobial Resistance Surveillance Network (EARS-Net) and the European Surveillance of Antimicrobial Consumption (4,5). Moreover, the Croatian Chapter of the Alliance for the Prudent Use of Antibiotics was founded in September 2002 (6), and in 2006 the Croatian Intersectoral Coordinating Mechanism (ISKRA) was established at the Croatian Ministry of Health and Social Welfare (4). This stringent network of surveillance proved to be a valuable information source for many European institutions that have a direct role in tracking AMR, interpreting and benchmarking data, and advocating for health policy updates – such as the European Centre for Disease Prevention and Control (ECDC) (7).

However, previous ECDC reports focused on prevalence surveillance of resistant invasive strains (ie, from cerebrospinal fluid and blood) and covered only eight bacterial species that were deemed of public health significance in Europe (8,9), an effort resulting in an incomplete epidemiologic representation. In 2015, research by Cassini et al (10) made an enormous leap forward from AMR prevalence monitoring to actual disease burden assessment with an analysis of European Union/European Economic Area (EU/EEA) countries, which measured case numbers, attributable mortality, and disability-adjusted life-years (DALYs) for eight pathogenic species and 16 pathogen-drug combinations. This work for the first time delivered several important and specific country-level insights (10).

Notwithstanding such an established history in resistance tracking and participation in the burden of AMR assessment, there is also an issue of whether the publication and dissemination of these results actually informed health care providers and influenced prescription patterns in Croatia. Croatia is placed among European countries that have a rather high total consumption of antimicrobial agents (albeit with somewhat decreasing trends recently), which, consequently, translates to high resistance rates of many pathogenic species (11). In Croatian hospitals, high rates of unwarranted antimicrobial prescriptions are observed, as well as the exaggerated use of broad-spectrum antibiotics (12). Likewise, a recent study on dental practitioners in Croatia demonstrated how broad-spectrum antimicrobial agents are frequently prescribed, even for indications where surgical approach is the preferred option (13). There are many more examples like these, foreshadowing the potential rise of a generation of highly resistant microorganisms that may translate to higher AMR mortality and disability.

Accordingly, there is a need for even better indicators of country-level AMR burden that would offer precise insights to health care professionals, the scientific community, and the general public to inform policy decisions. In this study, we aimed to present the most complete and wide-ranging country-level estimates of the bacterial AMR burden in 2019 for Croatia to date, showing the proportion of deaths and DALYs caused by antibiotic-resistant microorganisms across different infectious syndromes, and covering a comprehensive set of bacterial agents and pathogen-drug combinations with the use of two innovative counterfactual scenarios.

## Material and methods

This study used the methodological approach and robust estimation process of overall and age-specific deaths/DALYs from the global burden of AMR framework (2) to provide detailed and country-specific estimates for Croatia. In total, there were 471 million individual isolates or records (spanning across 7585 location-years) acquired in our comprehensive global data acquisition venture; data that primarily informed our estimation process for Croatia included detailed data sets comprising microbial data with and without outcome, single drug-resistance profiles, and systematic literature reviews.

More specifically, the majority of microbial data came from the SENTRY Antimicrobial Surveillance Program, Pfizer ATLAS Programme, European Antimicrobial Resistance Surveillance Network, Study for Monitoring Antimicrobial Resistance Trends, Global Antimicrobial Resistance Surveillance System by the World Health Organization (WHO), and specific country-level resistance data for tuberculosis and the Gonococcal Antimicrobial Surveillance Programme. More details on the data sources and systematic reviews that were used for the estimation process can be found in the Appendix of the article by Murray et al on the global burden of AMR (2), and data input records can be retrieved online (https://ghdx.healthdata.org/record/ihme-data/global-bacterial-antimicrobial-resistance-burden-estimates-2019).

We estimated the AMR disease burden for 12 main infectious syndromes and one residual group, 23 pathogenic bacteria, and 88 bug–drug combinations. Our overall approach consisted of five components: 1) mortality count where infection was implicated, 2) infectious deaths (proportion) that are attributable to a specific infectious syndrome, 3) infectious syndrome deaths (proportion) attributable to a specific pathogen, 4) pathogens that are resistant to an antimicrobial drug of interest in percentage terms, and 5) the excess death or infection duration risk associated with this resistance. These components enabled us to estimate disease burden based on two plausible counterfactual scenarios (counterfactuals): deaths that are attributable to AMR considering an alternative scenario where drug-resistant infections are substituted with sensitive ones, and deaths that are associated with AMR considering an alternative scenario where individuals with drug-resistant infections would instead be without any infection.

Across these five modeling components, there were ten broad estimation steps. Estimation steps 1-2 defined the death counts where infection was implicated by referring to the Global Burden of Disease (GBD) 2019 cause of death estimates (14), taking into account instances where there was an infectious underlying cause of death, or for which the pathway to death was via sepsis. Pathogen distributions of each infectious syndrome were estimated in steps 3-4, disjointedly for deaths and incident cases, while the prevalence of AMR by pathogen was ascertained in steps 5-7 via the analysis of the phenotypic AMR proportion for each of the pathogens within the 88 pathogen-drug combinations. In estimation steps 8-9, we calculated the relative risk of fatal outcome for every pathogen-drug combination for a resistant infection in comparison with the drug-sensitive one. This is where we also introduced a population-attributable fraction for every profile that showed resistance to at least one antibiotic as a metric that considered the prevalence of resistance, excess risk, and a redistribution of burden to each antibiotic class based on the respective excess risk (2). Finally, in estimation step 10, we computed the two mentioned counterfactuals to assess the AMR burden. Minimum inhibitory concentration breakpoints were defined according to Clinical and Laboratory Standard Institute guidelines (15) when these minimums were available.

### Statistical analysis

Advanced statistical techniques including the Bayesian meta-regression tool MR-BRT, a network analysis using multinomial estimation with partial and composite observations (by constructing a network model with the dependent variable as the log ratio of cases between different pathogens and estimated over a flexible parameterization of multinomial parameters using a maximum likelihood approach), a two-stage spatiotemporal modeling framework (to estimate the prevalence of resistance in each pathogen-drug combination), and spatiotemporal Gaussian process regression were used in the process. More details on our modeling approach and major methodological innovations can be found in the AMR global burden (2) and the burden of AMR in the WHO European Region publications (16), where our modeling framework is described in detail. Akin to GBD methods described previously (14), for each modeling step of the analysis we propagated uncertainty into the final estimates of burden attributable to or associated with drug resistance by reporting at the 2.5% and 97.5% of 1000 draws from the posterior distribution of our quantities of interest. This formed the foundation of our uncertainty intervals (UI). Finally, out-of-sample predictive validation was pursued for all the models as well.

## Results

In Croatia in 2019 there were 5333 deaths (95% UI 3378–8010) associated with one of 11 infectious syndromes (ie, those with lethal outcomes) as an underlying or an intermediate cause of death. Bacterial pathogens of interest (those we estimated antimicrobial resistance for) caused 4160 (2582–6237) of such deaths. The burden of associated and attributable mortality due to bacterial AMR was 2546 (1558–3803) and 614 (365–943) deaths, respectively. Among the estimated syndromes, bloodstream infections had the highest total burden with 1067 (529–1804) deaths associated with and 264 (132–460) deaths attributable to any resistant pathogen-drug combination, followed by peritoneal/intra-abdominal infections, infections of the urinary tract, and lower respiratory infections (by order of number of deaths). These four syndromes were behind more than 90% of fatal outcomes associated with and attributable to AMR in Croatia in 2019. A complete breakdown by infectious syndrome for deaths and DALYs is shown in Table 1.

**Table 1:** Overall antimicrobial resistance (AMR) burden by infectious syndrome in Croatia in 2019. We aggregated estimates across antimicrobial agents, taking into account the co-occurrence of resistance to more than one drug*

Infectious syndrome	Attributable to AMR				Associated with AMR			
	deaths		DALYs		deaths		DALYs	
	counts	rate per 100 000	counts	rate per 100 000	counts	rate per 100 000	counts	rate per 100 000
BSI	264 (132-460)	6.21 (3.10-10.83)	4950 (2483-8606)	116.51 (58.46-202-59)	1067 (529-1804)	25.11 (12.45-42-47)	20 035 (9922-33 683)	471.64 (233.58-792.94)
Bacterial skin infections	13 (3-33)	0.30 (0.07-0.77)	217 (52-565)	5.10 (1.22-13.31)	60 (14-152)	1.41 (0.33-3.57)	1016 (261-2583)	23.91 (6.14-60-81)
Bone and joint infections	2 (1-5)	0.05 (0.01-0.12)	39 (11-98)	0.92 (0.26-2.30)	10 (3-23)	0.23 (0.07-0.55)	179 (51-425)	4.22 (1.20-10.00)
CNS infections	2 (1-4)	0.05 (0.03-0.09)	59 (33-112)	1.39 (0.77-2.64)	9 (5-17)	0.21 (0.12-0.39)	273 (155-514)	6.42 (3.66-12.11)
Cardiac infections	25 (13-45)	0.59 (0.30-1.05)	422 (210-757)	9.92 (4.94-17.83)	102 (52-180)	2.41 (1.23-4.23)	1711 (863-3037)	40.28 (20.32-71.50)
Diarrhea	0.2 (0.1-0.5)	0.01 (0-0.01)	19 (8-36)	0.44 (0.19-0.84)	1 (0.5-2)	0.03 (0.01-0.05)	111 (50-201)	2.62 (1.18-4.74)
Gonorrhea and chlamydia†	–	–	1.5 (0.3-3)	0.03 (0.01-0.07)	–	–	14 (7-27)	0.33 (0.16-0.62)
Intra-abdominal infections	159 (95-247)	3.74 (2.24-5.81)	3273 (1902-5184)	77.05 (44.78-122.04)	666 (403-1015)	15.67 (9.49-23.90)	13 709 (8108-21 225)	322.73 (190.87-499.66)
LRI and thorax infections	71 (42-114)	1.68 (0.99-2.68)	1189 (680-1900)	27.99 (16.00-44.73)	293 (180-449)	6.89 (4.24-10-57)	4897 (2911-7648)	115.29 (68.53-180.04)
Tuberculosis	0.5 (0-1.4)	0.01 (0-0.03)	10 (1-29)	0.23 (0.03-0.68)	1.5 (1-3)	0.03 (0.01-0.06)	31 (15-62)	0.73 (0.34-1.46)
Typhoid, paratyphoid, and iNTS	0 (0-0)	0 (0-0)	0 (0-0)	0 (0-0)	0 (0-0)	0 (0-0)	0 (0-0)	0 (0-0)
UTI and pyelonephritis	77 (52-108)	1.81 (1.23-2.55)	1142 (769-1659)	26.89 (18.10-39.04)	337 (231-468)	7.93 (5.44-11.01)	4981 (3430-7075)	117.26 (80.74-166.55)
**Total**	**614 (364-943)**	**14.45 (8.59-22.19)**	**11 321 (6544-17 809)**	**266.50 (154.10-419.24)**	**2546 (1558-3803)**	**59.93 (36.68-89.52)**	**46 958 (28 033-71 628)**	**1105.44 (659.93-1686.21)**

*Abbreviations: BSI – bloodstream infections; CNS – meningitis and other bacterial central nervous system infections; LRI – lower respiratory infections and all related infections in the thorax; iNTS – intestinal nontyphoidal salmonellae; UTI – urinary tract infections and pyelonephritis; DALY - deaths/disability-adjusted life year.

†only DALY burden is presented as we did not estimate the fatal burden of the infectious syndrome.

For deaths associated with AMR, the overall crude and age-standardized mortality rates (ASMR) were 59.9 (95% UI 36.7–89.5) and 28.9 (17.4–43.6) per 100 000 people, respectively, while the overall crude and age-standardized associated DALY rates were 1105.4 (659.9–1686.2) and 629.7 (369.4–971.9) per 100 000 people, respectively. The burden of AMR among children younger than 5 years of age was low, accounting for only 0.34% and 1.67% of total infectious deaths and DALYs associated with bacterial resistance, respectively. Five pathogens dominated the landscape of mortality associated with AMR: *Escherichia coli* (694 [443–1017] deaths), *Staphylococcus aureus* (335 [198–522] deaths), *Acinetobacter baumannii* (288 [142–510] deaths), *Klebsiella pneumoniae* (281 [174–420] deaths), and *Pseudomonas aeruginosa* (210 [130–322] deaths) (Table 2). Each of these pathogens was found in more than 200 deaths, while *Enterococcus faecium*, *Streptococcus pneumoniae,* and *Enterobacter* spp caused more than one hundred deaths each (Table 2).

**Table 2:** Overall antimicrobial resistance (AMR) burden by pathogen in Croatia in 2019. We aggregated estimates across antimicrobial agents, taking into account the co-occurrence of resistance to more than one drug*

Pathogen	Attributable to AMR				Associated with AMR			
	deaths		DALYs^†^		deaths		DALYs	
	counts	rate per 100 000	counts	rate per 100 000	counts	rate per 100 000	counts	rate per 100 000
*Acinetobacter baumannii*	93 (45-165)	2.19 (1.07-3.88)	1680 (803-2972)	39.54 (18.90-69.97)	288 (142-510)	6.78 (3.52-6.78)	5193 (2534-9241)	122.25 (59.65-217.55)
*Citrobacter* spp	5 (2-11)	0.12 (0.05-0.26)	107 (46-227)	2.51 (0.95-5.33)	18 (8-36)	0.42 (0.18-0.85)	364 (151-738)	8.58 (3.56-17.38)
*Enterobacter* spp	29 (16-49)	0.69 (0.37-1.14)	588 (310-993)	13.83 (7.30-23.38)	123 (71-199)	2.89 (1.68-4.69)	2461 (1387-4045)	57.93 (32.65-95.23)
*Enterococcus faecalis*	26 (12-48)	0.61 (0.28-1.13)	506 (225-930)	11.91 (5.30-21.88)	99 (54-163)	2.33 (1.26-3.83)	1928 (1057-3141)	45.39 (24.88-73.94)
*Enterococcus faecium*	52 (27-88)	1.21 (0.63-2.08)	1036 (524-1794)	24.39 (12.35-42.22)	193 (113-293)	4.53 (2.67-6.89)	3868 (2227-5969)	91.05 (52.42-140.51)
*Escherichia coli*	148 (93-222)	3.48 (2.18-5.23)	2578 (1548-3927)	60.70 (36.43-92.43)	694 (443-1017)	16.33 (10.43-23.94)	12 159 (7503-18 325)	286.23 (176.63-431.39)
Group A *Streptococcus*	1 (0-3)	0.02 (0-0.07)	16 (0-59)	0.38 (0-1.38)	8 (3-16)	0.18 (0.07-0.38)	168 (76-331)	3.96 (1.78-7.79)
Group B *Streptococcus*	5 (0-13)	0.12 (0-0.32)	101 (0-273)	2.39 (0-6.42)	42 (24-70)	0.99 (0.56-1.65)	831 (468-1377)	19.58 (11.01-32.43)
*Klebsiella pneumoniae*	76 (43-122)	1.78 (1.02-2.86)	1368 (780-2228)	32.20 (18.36-52.45)	281 (174-420)	6.61 (4.10-9.89)	5090 (3068-7785)	119.82 (72.21-183.26)
Other enterococci	8 (2-17)	0.19 (0.04-0-41)	137 (31-298)	3.23 (0.72-7.01)	39 (21-67)	0.92 (0.50-1.58)	650 (331-1153)	15.30 (7.79-27.15)
*Proteus* spp	8 (4-14)	0.18 (0.09-0.32)	128 (61-229)	3.01 (1.43-5.40)	63 (41-91)	1.49 (0.97-2.15)	1040 (654-1528)	24.47 (15.42-35.98)
*Pseudomonas aeruginosa*	51 (30-81)	1.20 (0.71-1.91)	936 (540-1518)	22.04 (12.70-35.73)	210 (130-322)	4.95 (3.06-7.58)	3855 (2292-5980)	90.76 (53.95-140.76)
*Serratia* spp	2 (1-4)	0.05 (0.02-0.09)	43 (20-81)	1.01 (0.47-1.90)	8 (4-13)	0.18 (0.10-0.31)	163 (86-272)	3.84 (2.03-6.41)
*Staphylococcus aureus*	79 (39-142)	1.87 (0.91-3.34)	1512 (713-2724)	35.60 (16.79-64.12)	335 (198-522)	7.89 (4.66-12.30)	6393 (3712-10 152)	150.51 (87.39-238.98)
*Streptococcus pneumoniae*	29 (16-47)	0.68 (0.39-1.11)	553 (307-914)	13.02 (7.22-21.51)	139 (90-208)	3.28 (2.12-4.90)	2662 (1659-4078)	62.67 (39.05-96.00)
**All pathogens**	**614 (365-943)**	**14.45 (8.59-22.18)**	**11 321 (6544-17 809)**	**266.50 (154.06-419.24)**	**2546 (1558-3803)**	**59.93 (36.68-89.52)**	**46 958 (28 033-71 628)**	**1105.44 (659.93-1686.21)**

*Pathogens that did not contribute a significant burden (ie, that have two or fewer deaths associated with AMR) were not included in the table – namely, enteropathogenic and enterotoxigenic *E coli*, *Haemophilus influenzae, Morganella* spp, *Mycobacterium tuberculosis*, *Neisseria gonorrhoaeae, Salmonella* Typhi and Paratyphi, non-typhoidal salmonellae, and *Shigella* spp).

†DALY - deaths/disability-adjusted life year.

**Figure 1:**
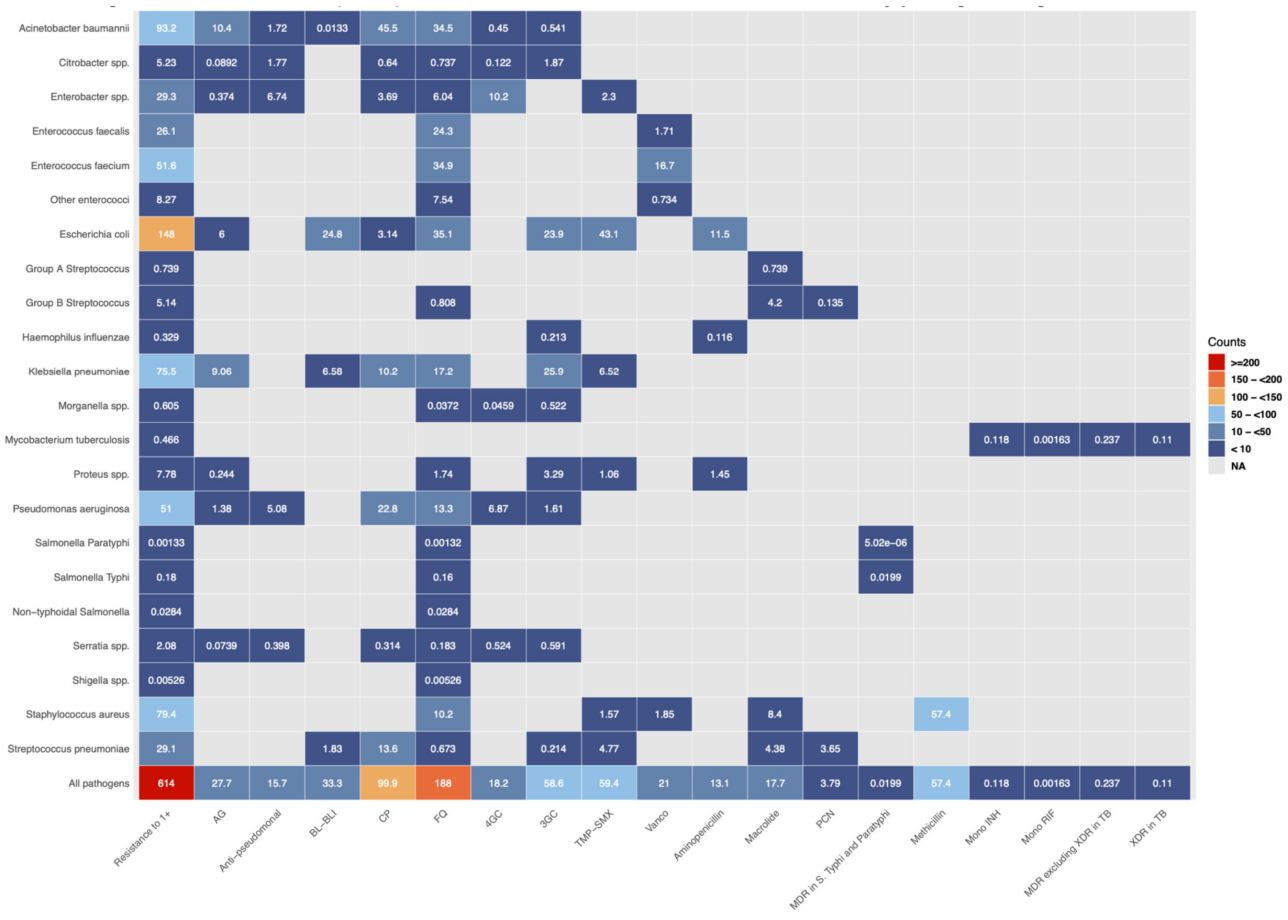
Death counts attributable to antimicrobial resistance (AMR) by pathogen-drug combination in Croatia in 2019. Abbreviations: 3GC - third-generation cephalosporins. 4GC - fourth-generation cephalosporins. Anti-pseudomonal - anti-pseudomonal penicillin or beta-lactamase inhibitors. BL-BLI - beta-lactam or beta-lactamase inhibitors. MDR - multidrug resistance. mono INH - isoniazid mono-resistance. mono RIF - rifampicin mono-resistance. NA - not applicable. Resistance to 1+ - resistance to one or more drug. TMP-SMX - trimethoprim-sulfamethoxazole. XDR - extensive drug resistance; AG - aminoglycosides; CP – carbapenems; FQ – fluoroquinolones; PCN – penicillin; TB – tuberculosis.

**Figure 2:**
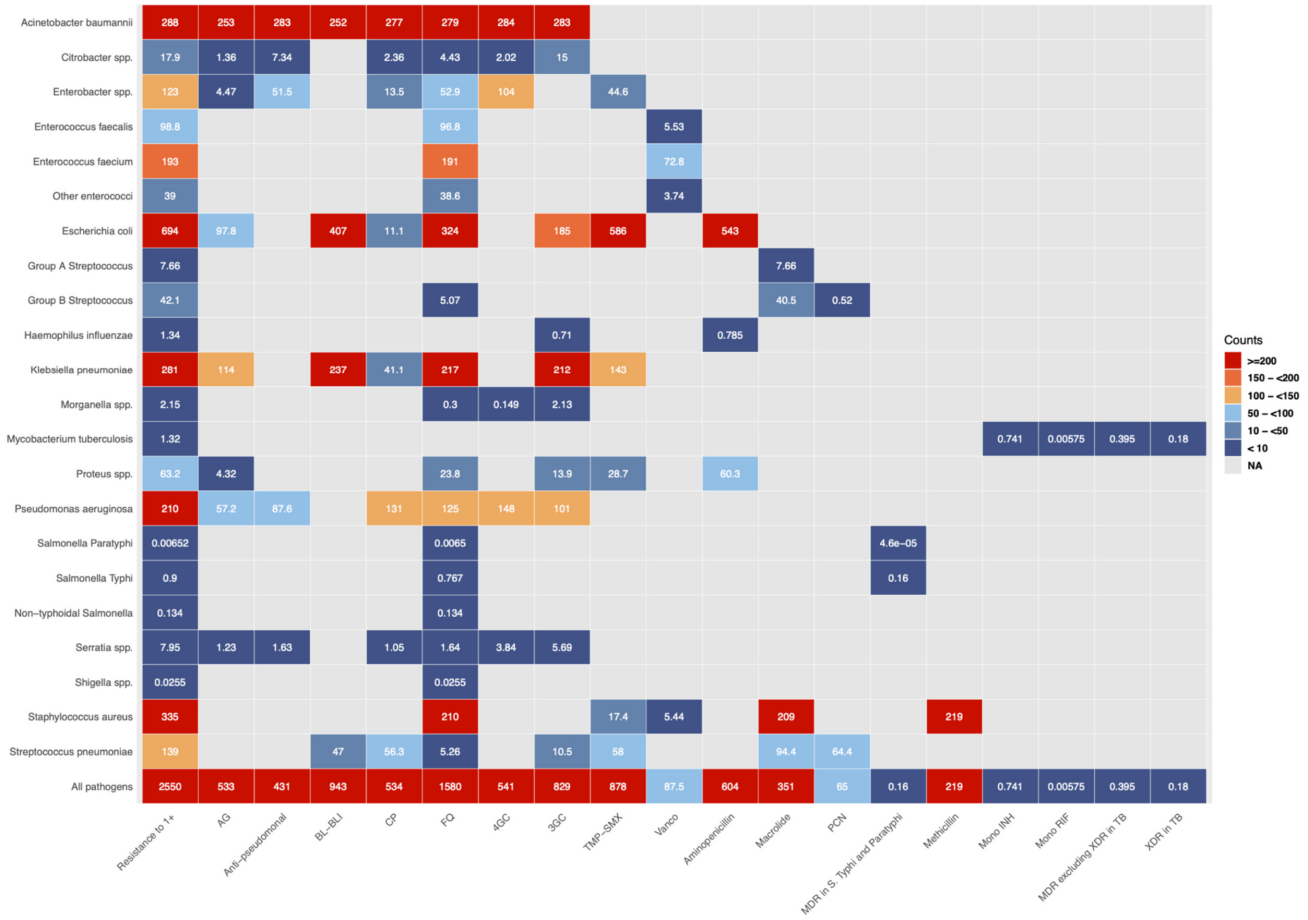
Death counts associated with antimicrobial resistance (AMR) by pathogen–drug combination in Croatia in 2019. Abbreviations: 3GC - third-generation cephalosporins. 4GC - fourth-generation cephalosporins. Anti-pseudomonal - anti-pseudomonal penicillin or beta-lactamase inhibitors. BL-BLI - beta-lactam or beta-lactamase inhibitors. MDR - multidrug resistance. mono INH - isoniazid mono-resistance. mono RIF - rifampicin mono-resistance. NA - not applicable. Resistance to 1+ - resistance to one or more drug. TMP-SMX - trimethoprim-sulfamethoxazole. XDR - extensive drug resistance; AG - aminoglycosides; CP – carbapenems; FQ – fluoroquinolones; PCN – penicillin; TB – tuberculosis.

**Figure 3:**
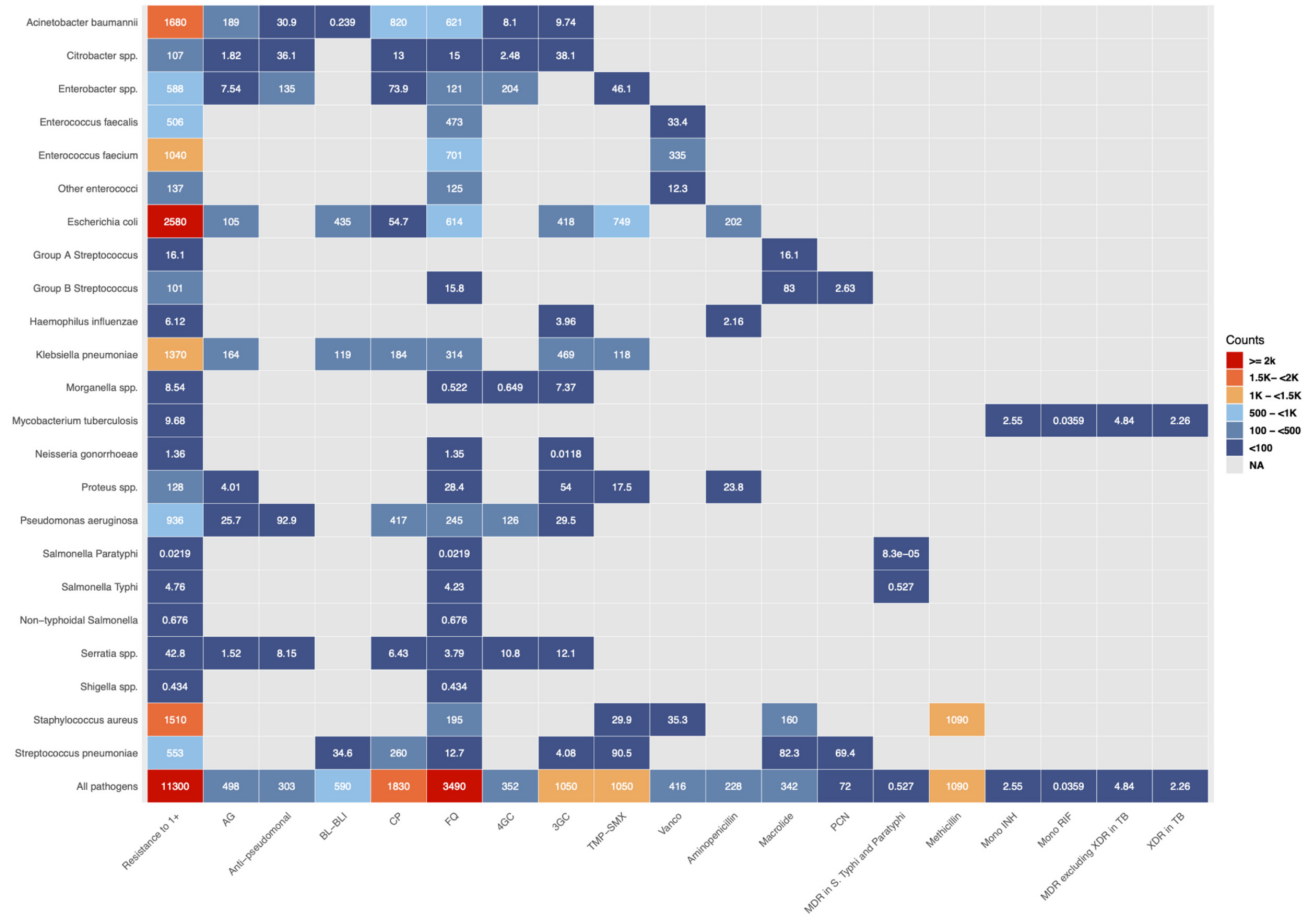
Disability-adjusted life year (DALY) counts attributable to antimicrobial resistance (AMR) by pathogen-drug combination in Croatia in 2019. Abbreviations: 3GC - third-generation cephalosporins. 4GC - fourth-generation cephalosporins. Anti-pseudomonal - anti-pseudomonal penicillin or beta-lactamase inhibitors. BL-BLI - beta-lactam or beta-lactamase inhibitors. MDR - multidrug resistance. mono INH - isoniazid mono-resistance. mono RIF - rifampicin mono-resistance. NA - not applicable. Resistance to 1+ - resistance to one or more drug. TMP-SMX - trimethoprim-sulfamethoxazole. XDR - extensive drug resistance; AG - aminoglycosides; CP – carbapenems; FQ – fluoroquinolones; PCN – penicillin; TB – tuberculosis.

**Figure 4:**
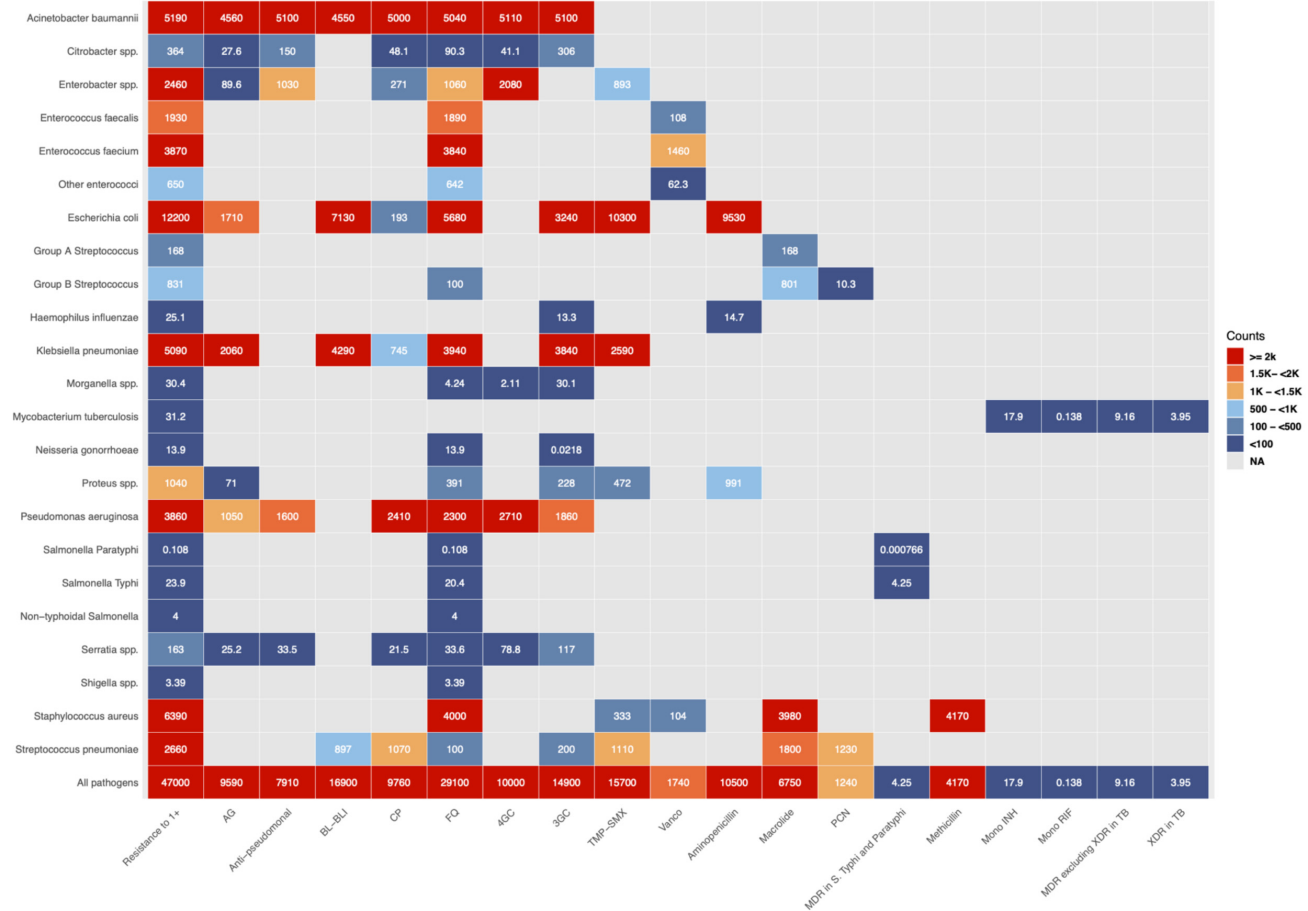
Disability-adjusted life year (DALY) counts associated with antimicrobial resistance (AMR) by pathogen-drug combination in Croatia in 2019. Abbreviations: 3GC - third-generation cephalosporins. 4GC - fourth-generation cephalosporins. Anti-pseudomonal - anti-pseudomonal penicillin or beta-lactamase inhibitors. BL-BLI - beta-lactam or beta-lactamase inhibitors. MDR - multidrug resistance. mono INH - isoniazid mono-resistance. mono RIF - rifampicin mono-resistance. NA - not applicable. Resistance to 1+ - resistance to one or more drug. TMP-SMX - trimethoprim-sulfamethoxazole. XDR - extensive drug resistance; AG - aminoglycosides; CP – carbapenems; FQ – fluoroquinolones; PCN – penicillin; TB – tuberculosis.

Regarding specific pathogen-drug combinations, methicillin-resistant *S. aureus* was the leading combination for the overall mortality attributable to AMR (57 [22–108] deaths; ASMR of 0.66 [0.26–1.25]), while *E coli* resistant to trimethoprim/sulfamethoxazole (co-trimoxazole) was responsible for most deaths associated with AMR (586 [374–861] deaths; ASMR of 6.48 [4.08–9.56] per 100 000) (Figures 1-2). These two pathogen-drug combinations were also leading for DALY burden attributable to and associated with AMR; more specifically, methicillin-resistant *S. aureus* and co-trimoxazole-resistant *E coli* were responsible for 1092 (421–2046) and 10 273 (6330–15,391) DALYs, respectively (Figures 3-4). When concentrating on specific infectious syndromes, the predominance of these two pathogen-drug combinations was observed in bloodstream infections, peritoneal/intra-abdominal infections, infections of the lower respiratory tract (together with related infections in the thorax), and urinary tract infections.

In addition to these two pathogen-drug combinations, carbapenem-resistant *A. baumannii*, fluoroquinolone-resistant *E coli*, and fluoroquinolone-resistant *E. faecium* (ordered sequentially in accordance with burden magnitude) were among the top pathogen-drug combinations by attributable AMR burden for each infectious syndrome. In addition to co-trimoxazole-resistant *E coli*, there were three more pathogen-drug combinations among the leading combinations of associated AMR burden (both deaths and DALYs) that included *E coli*: those resistant to aminopenicillin, beta-lactam/beta-lactamase-inhibitor (co-amoxiclav), and fluoroquinolones. Interestingly, these three specific pathogen-drug combinations had comparatively different fatal and nonfatal burdens when assessed across infectious syndromes, as the highest number of deaths (jointly) was estimated for bloodstream infections (471 in total), whereas the highest number of DALYs (jointly) was estimated for peritoneal and intra-abdominal infections (8175 in total).

## Discussion

To our knowledge, this is the most detailed study thus far that reports the burden of mortality and DALYs due to AMR for Croatia, and the first one to use two measures – burden attributable to and associated with AMR – for a wide-ranging list of pathogens and pathogen-drug combinations. We demonstrated that bacterial resistance played a considerable role in excess mortality in 2019, as more than 2500 deaths could have been fended off if all resistant infections had been substituted by no infection, whereas more than 600 lives could have been saved if all drug-resistant infections had been substituted by drug-sensitive ones. These exhaustive, country-level estimates are novel and, thus, hold immense value for progress in this field.

In their seminal research systematically evaluating the antibiotic resistance burden in the EU, Cassini et al (10) estimated 240 (95% CI 204–278) deaths (5.67 rate per 100 000) and 7714 (6667–8837) DALYs (182.56 rate per 100 000) for Croatia in 2015 for their 16 pathogen-drug combinations. This is lower than our estimates for 2019 – even attributable ones, which we consider as a more conservative number. A total of 11 pathogen-drug combinations overlap between the study by Cassini et al (10) and our study. Specifically, we did not pursue colistin resistance estimation in *E coli*, *P. aeruginosa*, or *A. baumannii* as a result of data scarcity regarding colistin resistance (which is a current concern for Europe in general), while multidrug resistance (MDR) estimation in *P. aeruginosa* or *A. baumannii* was not assessed due to our approach to MDR infections (2). For the 11 overlapping combinations, we estimated 260 deaths and 4781 DALYs attributable to AMR, as well as 1846 deaths and 33 785 DALYs associated with AMR, compared with Cassini et al estimates (10) that showed 236 deaths and 7573 DALYs linked to resistance. While these values are comparable (particularly with respect to the attributable scenario), any interpretation and policy uptake of estimates should consider methodological differences of these studies and those of others to preserve clarity and transparency. Alongside methodological differences, year of estimation, and a larger number of sources, we covered a much larger number of pathogen-drug combinations and, for the first time, took into account two different counterfactual scenarios to provide a sense of the upper and lower bounds of AMR burden.

Studies conducted in Croatia in recent years show some interesting trends that go hand in hand with our burden estimates. For example, antibiotic consumption surveillance data for Croatia in 2019 indicate that co-amoxiclav was the most commonly prescribed antimicrobial agent in outpatient settings, while amoxicillin was second (17,18). Relatedly, *E coli* resistant to beta-lactam/beta-lactamase-inhibitor and aminopenicillin was among the top three pathogen-drug combinations for the burden associated with AMR in our study. Likewise, an 8% increase in fluoroquinolone usage in the outpatient setting in Croatia over 2019 (9) correlates with the high burden of fluoroquinolone-resistant *E coli* in our study for both attributable and associated AMR burden. Resistance in uropathogenic *E coli* is recognized as a threat on a national level (19,20), while fluoroquinolone resistance in urinary *E coli* isolates increased by 54.5% between 2007 and 2014 (21).

Our results support the established significance of methicillin-resistant *S. aureus* (MRSA), particularly in bloodstream infections, where this pathogen-drug combination was responsible for 28 (10–61) and 109 (53–190) deaths in 2019 attributable to and associated with AMR, respectively. After 2008, MRSA rates decreased, with resistance rates falling to 12% by 2014; however, since 2015 MRSA rates started to increase again and reached 16% in 2019 (17). Our estimates identified carbapenem-resistant *A. baumannii* as the second most important pathogen-drug combination for deaths and DALYs attributable to AMR. This is of no surprise, as *A. baumannii* resistance to carbapenems surged in Croatia after 2008 and now regularly surpasses 80% in yearly surveillance reports – reaching 90% in 2019 (17). Conversely, a relatively smaller burden of *S. pneumoniae* in our study (when compared with other pathogens) may be explained by the use of the higher-valent pneumococcal vaccines, which substantially reduced the prevalence of multidrug-resistant strains, and in turn the rate of invasive pneumococcal disease (22).

Resistant strains of *K. pneumoniae* ranked fourth in our overall estimated burden associated with AMR. Historically implicated in health care-associated infections, *K. pneumoniae* has been identified as a pathogen of growing importance and an independent risk factor for bacteremia-related fatal outcomes (23,24). A recent study from Zagreb County in Croatia highlighted an eruptive and extensive spread of carbapenem-resistant *K. pneumoniae* strains in long-term care facilities and the community, particularly in individuals with infections of the urinary tract and those with urinary catheters (25). In our study, resistant *K. pneumoniae* strains were responsible for 43 deaths and 631 DALYs within urinary tract infections, substantially contributing to the total envelope. With respect to carbapenem-resistant *K. pneumoniae*, we found that its contribution to the AMR burden in urinary tract infections was 14.7% and 14.6% for deaths and DALYs, respectively. An extensive molecular analysis of strains from three distinct regions in Croatia revealed that the majority of carbapenemase-producing *K. pneumoniae* isolates also demonstrated resistance to gentamicin (26).

Gram-negative bacteria dominated the resistance burden in Croatia; however, *E. faecium* gained prominence as a paramount nosocomial pathogen that contributed significantly to AMR mortality. The first report that explored features of vancomycin-resistant *Enterococcus* strains in Croatia highlighted the propensity of horizontal gene transfer, but also potential resistance transfer to other bacterial pathogens like *S. aureus*, which may further complicate the resistance landscape (27). A recent study by Todorić et al (28) showed an increasing trend in enterococcal bacteremia in Croatia and a significantly higher proportion of vancomycin resistance, with patients in intensive care units being most vulnerable to such infections. Interestingly, our estimates indicate that fluoroquinolone-resistant *E. faecium* is a more frequent pathogen-drug combination than its vancomycin-resistant counterpart. In our study, deaths and ASMRs attributable to AMR for *E. faecium* resistant to vancomycin were 17 (8–30) and 0.19 (0.09–0.36), respectively, and those associated with AMR were 73 (42–117) and 0.84 (0.48-1.37), respectively. However, these metrics were much higher for *E. faecium* resistant to fluoroquinolones, with 35 (10–68) deaths and 0.40 (0.11-0.79) ASMR attributable to AMR, as well as 191 (113–290) deaths and 2.21 (1.28-3.4) ASMR associated with AMR.

This is another warning signal about the importance of adequately addressing fluroquinolone prescription habits in Croatia. A recent survey involving 12 urology departments in Croatia showed that fluoroquinolones were by far the most commonly prescribed class of antibiotics (accounting for 84% of prescriptions), and the authors have emphasized how the lack of uniform guidelines contributes to varying protocols, ultimately leading to a concerning escalation in antibiotic resistance (29).

We would like to state several limitations of this study, primarily a relatively low number of microbiological data points linked to clinical outcomes; this can be viewed as a general problem that will have to be addressed by improving and expanding systematic data collection approaches (30). Additionally, other important drug-resistant microorganisms have to be included to observe the full scope of the problem – not only bacteria, but also fungi, protozoan parasites, and even viruses. We accounted for many potential biases in our methodological approach, but our findings may be influenced by some selection biases within passive microbial surveillance data. Combining and standardizing data from diverse providers, particularly when distinguishing between community-acquired and health care-associated infections, introduces potential sources of misclassification. Also, there is a need to incorporate more country-level sources in future analyses to enhance the precision and relevance of the model outcomes. Notwithstanding these limitations, our country-level analysis can be viewed as the most comprehensive investigation of the bacterial resistance burden for Croatia to date, utilizing the best currently available data sets, as well as the models that were implemented and perfected specifically for incorporating disparate data sources for the GBD analysis.

In conclusion, we showed that the burden of AMR in Croatia was considerable, which means there is a need for a rigorous systems approach that will address the complexities and nuance surrounding AMR as a public health threat. Although Croatia is committed to meticulous resistance tracking, tireless monitoring is of utmost importance (together with antibiotic consumption and health outcomes investigations) in order to close the gap between research and policy. National data serve as the foundation for monitoring trends, evaluating interventions, and guiding decision-making to effectively address the challenges posed by AMR. Without consistent and accurate data, the pursuit of research endeavors in this critical field would be hindered. This makes it crucial for countries like Croatia to prioritize and invest in enhancing their data-collection capabilities. We believe that our estimates represent an important step toward that goal.
